# Impact of right ventriculotomy for tetralogy of Fallot repair with a pulmonary valve–sparing procedure

**DOI:** 10.1016/j.xjon.2021.10.061

**Published:** 2022-01-22

**Authors:** Yoshikazu Ono, Takaya Hoashi, Kenta Imai, Naoki Okuda, Motoki Komori, Kenichi Kurosaki, Hajime Ichikawa

**Affiliations:** a Department of Pediatric Cardiovascular Surgery, National Cerebral and Cardiovascular Center, Suita, Osaka, Japan; b Department of Pediatric Cardiology, National Cerebral and Cardiovascular Center, Suita, Osaka, Japan

**Keywords:** inverse probability weighting, pulmonary valve-sparing procedure, right ventriculotomy, tetralogy of Fallot, CI, confidence interval, HR, hazard ratio, IQR, interquartile range, PVS, pulmonary valve sparing, TAP, transannular patch, TOF, tetralogy of Fallot, VSD, ventricular septal defect

## Abstract

**Objectives:**

The study objectives were to reconfirm the superiority of the pulmonary valve-sparing procedure versus the transannular patch procedure for repair of tetralogy of Fallot and to evaluate the influence of a right ventriculotomy in the pulmonary valve-sparing procedure.

**Methods:**

Between 1978 and 2003, 440 patients (aged <10 years) underwent tetralogy of Fallot repair. Of these patients, 242 (55.0%) underwent the transannular patch procedure, 106 (24.1%) underwent the pulmonary valve-sparing procedure without right ventriculotomy, and 92 (20.9%) underwent the pulmonary valve-sparing procedure with right ventriculotomy. End points focused on adverse events and included all-cause mortality, reoperation, catheter intervention, and symptomatic arrhythmias. To compare the outcomes of pulmonary valve sparing with and without right ventriculotomy, inverse probability weighting was applied to adjust for potential confounding factors.

**Results:**

The median follow-up period was 20.3 years (interquartile range, 10.7-27.6). In all cohorts, the pulmonary valve-sparing procedure was the independent factor that reduced adverse events after tetralogy of Fallot repair (hazard ratio, 0.47; 95% confidence interval, 0.23-0.94; *P* = .033). After weighting, there was no difference in overall survival or event-free survival in the pulmonary valve-sparing with and without right ventriculotomy group. However, the pulmonary valve-sparing with right ventriculotomy group exhibited a larger cardiothoracic ratio (beta: 6.01; 95% confidence interval, 2.36-9.66; *P* = .001), lower medication-free rate (odds ratio, 0.29; 95% confidence interval, 0.098-0.79; *P* = .019), and higher New York Heart Association functional classification (odds ratio, 2.99; 95% confidence interval, 1.36-6.80; *P* = .007) at the latest follow-up.

**Conclusions:**

Right ventriculotomy for tetralogy of Fallot repair with pulmonary valve-sparing did not increase major adverse events. However, negative impacts on current status cannot be ignored.

## Graphical abstract

The comparison between TOF repair by PVS with and without right ventriculotomy showed no significant differences in overall survival, event-free survival, and pulmonary valve regurgitation-free rates, but the PVS without right ventriculotomy group showed a smaller cardiothoracic ratio, superior New York Heart Association functional status, and less medication.

**Figure:**
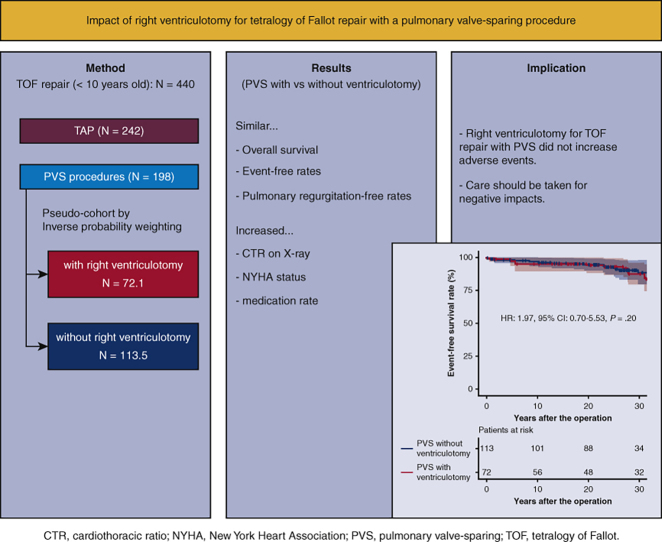

**Figure:**
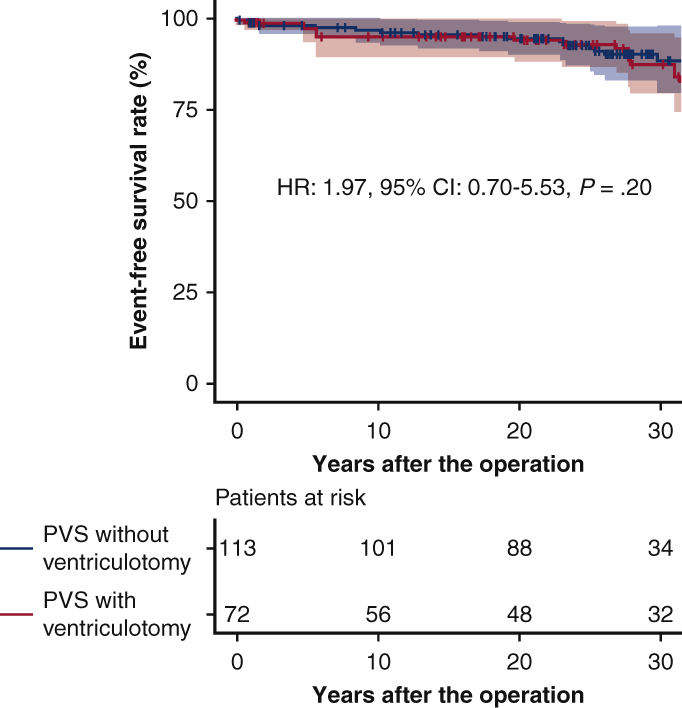
Event-free survival in the weighted cohort for PVS procedures. Bands above and below the fitted line represent 95% CIs.

**Central Message**

A right ventriculotomy for TOF repair with a PVS procedure does not increase major adverse events, but negative impacts on recent clinical status cannot be ignored.

**Perspective**

The pulmonary valve should be spared during TOF repair even if a right ventriculotomy is required because it has a proven protective effect against late adverse events. Both valve-sparing procedures showed similar good overall outcomes, but right ventriculotomy is associated with some negative impacts on late clinical status; to date, these are subclinical, but they are definitely present.

In addition to the closure of a large malaligned ventricular septal defect (VSD), relieving a right ventricular outflow tract obstruction is an important technical component in the repair of tetralogy of Fallot (TOF).^1^, ^2^, ^3^ Because there are currently no permanent competent valve prostheses with growth potential, the native pulmonary valve annulus and leaflets should be spared as much as possible to avoid pulmonary regurgitation. Indeed, late right ventricular dysfunction derived from long-standing ventricular volume overload by pulmonary regurgitation after TOF repair has been frequently observed.^4^, ^5^, ^6^

A right ventriculotomy is sometimes required during TOF repair with a pulmonary valve-sparing (PVS) procedure to complete an infundibulectomy and to enlarge the native pulmonary valve area.^7^, ^8^, ^9^ However, there are negative effects associated with a right ventriculotomy, such as impairment of right ventricular contraction, aneurysmal dilatation, or arrhythmogenicity.^4^^,^^10^, ^11^, ^12^

Our institute previously reported superior long-term outcomes of a TOF repair with a PVS procedure without right ventriculotomy.^13^ To date, however, no comparative study between PVS with and without right ventriculotomy has been possible because different operative indications had been adopted in different surgical time periods by different surgeons (Figure E1). Now, a recently introduced statistical modification of propensity score matching, referred to as the “inverse probability weighting method,” has made it possible to conduct this analysis.^14^

Therefore, the secondary objective of this study was to review the long-term outcomes of TOF repair to reconfirm the superiority of the PVS procedure compared with a transannular patch (TAP). The main objective was to reveal the effects of a right ventriculotomy in PVS procedures using the inverse probability weighting method.

## Materials and Methods

### Ethical Statement

The Institutional Review Board at the National Cerebral and Cardiovascular Center approved this retrospective study (R19043, 24/3/2020). Opt-out consent was obtained from the patients or parents/guardians of the patients instead of obtaining individual written informed consent.

### Patients

**Figure 1:**
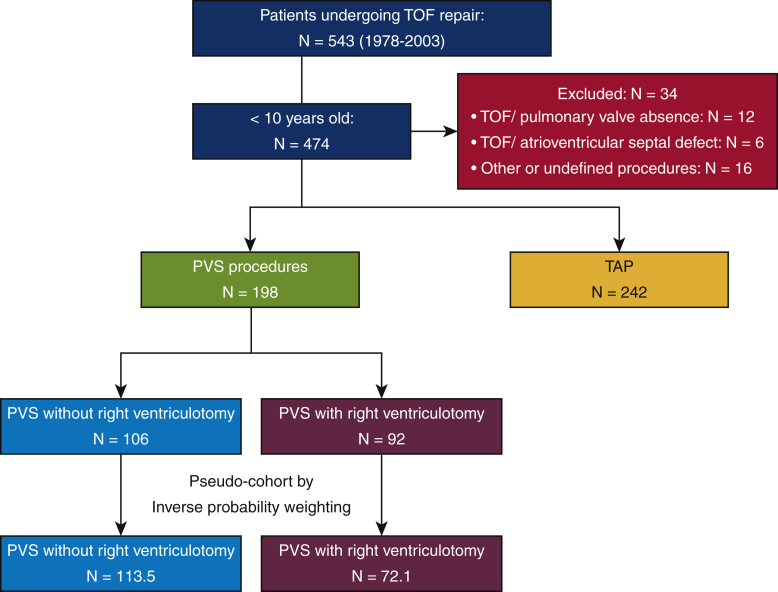
Flow chart showing selection of patients for this study. *TOF*, Tetralogy of Fallot; *PVS*, pulmonary valve sparing; *TAP*, transannular patch.

Of the 543 patients who underwent TOF repair from 1978 to 2003, 440 patients aged less than 10 years at the time of their surgery were enrolled (Figure 1). Patients with an absent pulmonary valve (n = 12), with an atrioventricular septal defect (n = 6), or whose defects were repaired using another or an undefined procedure (n = 16) were excluded. Among the remaining patients, 242 (55.0%) had undergone TAP and 198 (45.0%) had undergone the PVS procedure. Inverse probability weighting was applied, and a pseudo-cohort was created for the PVS group. This cohort was split into 2 groups: PVS with right ventriculotomy group (72.1 patients) and PVS without right ventriculotomy group (113.5 patients).

### Transition Between Surgical Procedures

**Figure E1:**
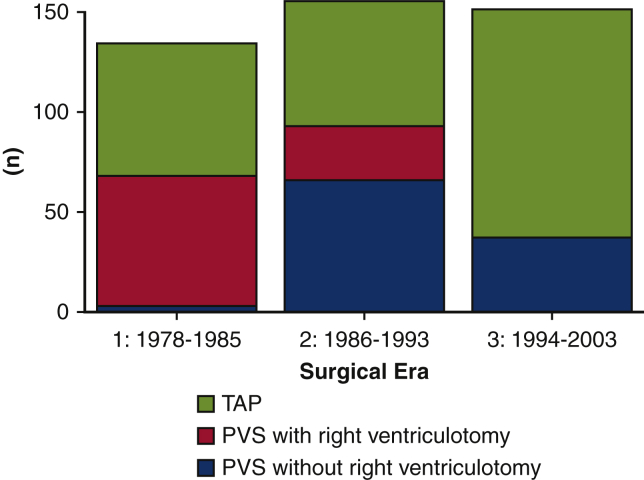
Trend in surgical procedures. *TAP*, Transannular patch; *PVS*, pulmonary valve sparing.

At the beginning of the study period, TOF repair was mainly carried out by means of a TAP or PVS with right ventriculotomy (Figure E1). Only a few noncyanotic, “pink” patients underwent PVS without right ventriculotomy, also referred to as a “transpulmonary and atrial repair.” In the mid-1980s, a new surgical team replaced the former team and the institutional strategy shifted from PVS with right ventriculotomy to PVS without right ventriculotomy (Video 1). This change in strategy occurred because at that time, a right ventriculotomy was believed to have an adverse impact on right ventricular function later in life. However, there were some patients with moderate pulmonary stenosis whose right ventricular outflow tract obstruction remained even after transpulmonary and atrial repair such that they needed a reoperation. Since 1990, these patients have undergone TAP with right ventriculotomy a few millimeters in length. This procedure, also referred to as a “minimal ventriculotomy,” was used because at the time this decision was made, an annulus division made with a ventricular incision that was a few millimeters long was not believed to impair right ventricular or pulmonary valvular function. As a result, a transannular approach was more frequently used in the later period of this study.

### Study Methods

The end points of the study were set as adverse events including all-cause mortality, reoperation, and arrhythmias that required intervention or hospitalization. First, the overall survival, event-free survival, and moderate or greater pulmonary regurgitation-free rates were evaluated between the PVS and TAP groups. Additionally, a risk analysis for event-free survival was conducted. Next, the risk factors for adverse events were evaluated in all cohorts.

To evaluate the impact of right ventriculotomy in PVS procedures, survival, event-free survival, and moderate or greater pulmonary regurgitation-free rates were compared in the pseudo-cohort groups of PVS with or without right ventriculotomy. Furthermore, current follow-up data including echocardiography, electrocardiography, New York Heart Association classification, serum brain natriuretic peptide level, and medication status were also compared.

The continuous variables were presented as the mean with standard deviation or as the median with interquartile range (IQR). The categorical variables were presented as counts and percentages. The logistic regression model was used to estimate the propensity score of patients in the PVS with right ventriculotomy group. The model included age, body weight, number of pulmonary valve leaflets, staged operation, Z-scores for pulmonary valve diameter, pulmonary artery index,^15^ genetic condition, and surgical era. The stabilized inverse probability weighting approach was used to create the pseudo-population.^16^ Balance was assessed with the standardized mean difference approach, and a standardized mean difference less than 0.25 was considered an acceptable balance.

The Mann–Whitney *U* test, chi-square test, and Fisher exact test were used to analyze between-group comparisons. The unweighted or weighted Cox proportional hazards regression model and the unweighted or weighted Kaplan–Meier method were used to evaluate the survival rates or risk factors for event-free survival. All statistical analyses were performed using R 4.0.3 (The R Foundation for Statistical Computing, Vienna, Austria).

## Results

The characteristics of each patient group are summarized in Table 1. Age, body weight, and body surface area at operation, probability of patients undergoing staged repair, and patients with bicuspid pulmonary valve were different between groups. The median pulmonary valve diameter was 8.9 mm (IQR, 7.0-10.0) in the TAP group and 12.0 mm (IQR, 11.0-14.0) in the PVS group (*P* < .001). The median Z-score for the pulmonary valve diameter, which was calculated using a previously published model,^17^ was −1.89 (IQR, −3.11 to −0.95) in the TAP group and 0.05 (IQR, −0.39 to 0.66) in the PVS group (*P* < .001).

**Table 1:** Patient characteristics

Group	TAP	PVS	*P*
No. of patients	242	198	
Female	101 (41.7)	78 (39.4)	.63
Age at repair (y)	1.6 [0.9, 2.5]	1.9 [1.3, 2.7]	.001
Body weight at repair (kg)	9.3 [7.9, 11.7]	10.4 [8.7, 12.1]	.002
Body surface area (m^2^)	0.43 [0.38, 0.52]	0.46 [0.41, 0.53]	.004
Surgical era			<.001
1978-1985	66 (27.3)	68 (34.3)	
1986-1993	62 (25.6)	93 (47.0)	
1994-2003	114 (47.1)	37 (18.7)	
Follow-up period (y)	19.3 [11.0, 26.5]	23.2 [16.3, 29.2]	.002
Staged repair	67 (27.7)	20 (10.1)	<.001
Chromosome anomaly			
Trisomy 21	8 (3.3)	6 (3.0)	1.00
22q11.2 deletion	4 (1.7)	1 (0.5)	.39
Others	2 (0.8)	0 (0.0)	.50
Subdiagnosis			
Patent ductus arteriosus	11 (4.5)	5 (2.5)	.31
Patent foramen ovale	97 (40.1)	81 (40.9)	.92
Morphology of pulmonary valve (n = 411, 93.4%)			<.001
Unicuspid	16 (7.4)	2 (1.0)	
Bicuspid	179 (83.3)	137 (69.9)	
Tricuspid	20 (9.3)	57 (29.1)	
Pulmonary artery measurements			
Modality (n = 406, 92.3%)			<.001
Catheter angiography	145 (69.7)	89 (44.9)	
Intraoperative measurement	63 (30.3)	109 (55.1)	
Pulmonary valve (n = 406, 92.3%) (mm)	8.9 [7.0, 10.0]	12.0 [11.0, 14.0]	<.001
Z score for pulmonary valve (n = 367, 83.4%)	-1.89 [-3.11, -0.95]	0.05 [-0.39, 0.66]	<.001
Right pulmonary artery (n = 293, 66.6%) (mm)	8.9 [7.4, 10.2]	9.4 [8.0, 10.3]	.052
Left pulmonary artery (n = 282, 64.1%) (mm)	8.0 [6.7, 10.0]	8.7 [7.3, 10.0]	.043
Pulmonary artery index (n = 275, 62.5%) (mm^2^/m^2^)	279 [202, 384]	274 [223, 357]	.73
Perioperative characteristics			
Aortic crossclamping time (min)	92 [75, 107]	85 [73, 99]	.064
Cardiopulmonary bypass time (min)	183 [145, 222]	156 [132, 178]	<.001
Right ventriculotomy length (mm)	15 [10, 20]	24 [20, 25]	<.001
Longitudinal right ventricular length (mm)	65 [58, 70]	65 [60, 70]	.61
Approach for VSD			.246
Right ventricle	116 (49.6)	86 (43.9)	
Right atrium/pulmonary artery	118 (50.4)	110 (56.1)	
Branch pulmonary artery plasty	31 (12.8)	5 (2.5)	<.001
Monocusp for TAP	165 (83.8)	-	
Autologous pericardium	53 (22.3)	-	
Expanded polytetrafluoroethylene	26 (10.9)	-	
Swine pericardium	61 (25.6)	-	
Equine pericardium	16 (6.7)	-	
Bovine pericardium	3 (2.1)	-	
Postoperative RVOT (mm)	12.0 [12.0, 13.0]	12.0 [11.0, 13.0]	.033
sRVP/ABP at operation	0.60 [0.50, 0.71]	0.55 [0.45, 0.64]	.001

Data represented as number (%) or median [25th, 75th percentile]. *TAP*, Transannular patch; *PVS*, pulmonary valve-sparing; *VSD*, ventricular septal defect; *RVOT*, right ventricular outflow tract; *sRVP*, systolic right ventricular pressure; *ABP*, arterial blood pressure.

### Perioperative Characteristics

All procedures were performed under mild hypothermic cardiopulmonary bypass and cardioplegic arrest with an antegrade crystalloid cardioplegic solution infusion. Perioperative characteristics are summarized in Table 1. The median cardiopulmonary bypass time was 183 minutes (IQR, 145-222) for the TAP group and 156 minutes (IQR, 132-178) for the PVS group (*P* < .001). The VSD was closed solely via the right atrium in 118 patients (50.4%) in the TAP group and 101 patients (51.5%) in the PVS group. The median ratio of the systolic right ventricular pressure to the arterial blood pressure, after weaning from cardiopulmonary bypass at operation, was 0.60 (IQR, 0.50-0.71) for the TAP group and 0.55 (IQR, 0.45-0.64) for the PVS group (*P* = .001). The diameter of the right ventricular outflow tract was measured using Hegar dilators after reconstruction. The median postoperative right ventricular outflow tract diameter was 12.0 mm (IQR, 12.0-13.0) in the TAP group and 12.0 mm (IQR, 11.0-13.0) in the PVS group (*P* = .033). For the TAP, a handmade monocusp was attached in 165 patients (83.8%). The monocusp was made of porcine pericardium for 61 patients (25.6%), autologous pericardium for 53 patients (22.3%), extended polytetrafluoroethylene for 26 patients (10.9%), equine pericardium for 16 patients (6.7%), and bovine pericardium for 3 patients (2.1%).

### Overall Outcomes

**Figure 2:**
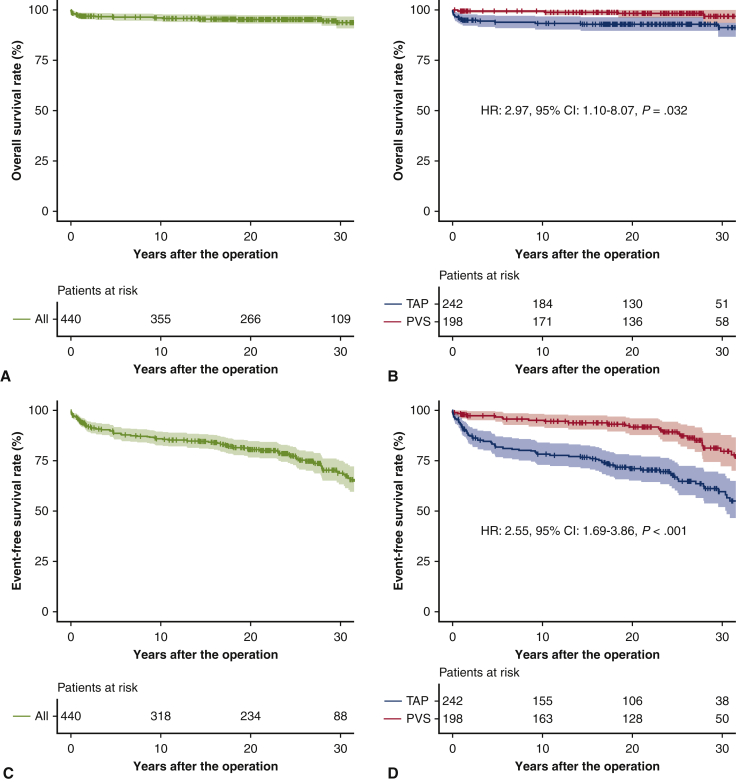

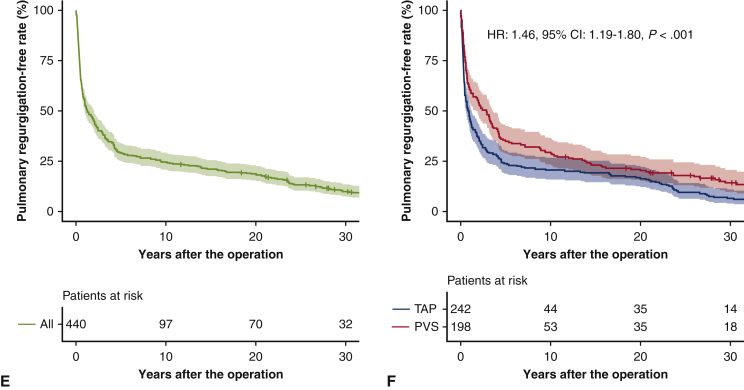
Overall survival in all study cohorts (A) and by procedures (B). Adverse event-free survival in all cohorts (C) and by procedures (D). Moderate or greater pulmonary regurgitation-free rates in all study cohorts (E) and by procedures (F). *Bands above and below the fitted line* represented 95% CIs. *HR*, Hazard ratio; *CI*, confidence interval; *TAP*, transannular patch; *PVS*, pulmonary valve sparing.

Overall follow-up rates in all study cohorts were 80.7% (355/440 patients) at 10 years, 60.5% (266/440) at 20 years, and 24.8% (109/440) at 30 years. Currently, 258 of 440 patients (58.6%) are being followed up at our institute. The overall survivals at 10, 20, and 30 years were 99.5%, 98.2%, and 96.8% in the PVS group, and 93.4%, 92.9%, and 91.2% in the TAP group, respectively (hazard ratio [HR], 2.97; 95% confidence interval [CI], 1.10-8.07; *P* = .032) (Figure 2, *A*, *B*). In the TAP group, 77 patients developed an adverse event: 17 mortalities, 13 atrial arrhythmia cases, 6 ventricular arrhythmia cases, 3 permanent pacemaker cases, 9 pulmonary valve replacement cases, 13 redo right ventricular outflow tract reconstruction cases, and 35 catheter intervention cases. In the PVS group, 32 patients developed adverse events: 5 mortalities, 8 atrial arrhythmia cases, 5 ventricular arrhythmia cases, 5 permanent pacemaker cases, 2 pulmonary valve replacement cases, 8 redo right ventricular outflow tract reconstruction cases, and 8 catheter intervention cases. The event-free survivals at 10, 20, and 30 years were 95.1%, 91.8%, and 79.7% in the PVS group, and 78.3%, 71.2%, and 59.8% in the TAP group, respectively (HR, 2.55; 95% CI, 1.69-3.86; *P* < .001) (Figure 2, *C*, *D*). Moderate or greater pulmonary regurgitation-free rates at 10, 20, and 30 years were 29.3%, 20.3%, and 14.2% in the PVS group, and 20.6%, 16.4%, and 6.6% in the TAP group, respectively (HR, 1.46; 95% CI, 1.19-1.80; *P* < .001) (Figure 2, *E*, *F*).

### Risk Factors for Postoperative Events

Table 2 presents the univariate and multivariate analyses of the risk factors affecting event-free survival after TOF repair using the Cox proportional hazards model. Age, sex, body weight, genetic condition, surgical era, staged repair, Z-scores for pulmonary valve diameter, right ventriculotomy, and PVS procedure were found to be significant risk factors in the univariate analysis. Of the factors presented, only the TAP procedure was detected as a risk factor by multivariate analysis (HR, 0.47; 95% CI, 0.23-0.94; *P* = .033).

**Table 2:** Multivariate analyses of the risk factors on event-free survival after tetralogy of Fallot repair

Model	Univariate			Multivariate		
	HR (95% CI)		*P*	HR (95% CI)		*P*
Age	0.85 (0.73-1.00)		.046	1.04 (0.77-1.39)		.81
Sex (male)	1.19 (0.81-1.75)		.37			
Body weight (kg)	0.89 (0.83-0.96)		.003	0.89 (0.77-1.03)		.10
Genetic condition	1.20 (0.53-2.69)		.22			
Surgical era						
1978-1985	1 (-)		-			
1986-1993	1.63 (0.99-2.71)		.056			
1994-2003	2.19 (1.27-3.78)		.005	1.04 (0.60-1.82)		.88
Staged repair	1.65 (1.07-2.54)		.023	1.22 (0.72-2.08)		.47
Z score for pulmonary valve diameter	0.86 (0.77-0.95)		.004	0.96 (0.83-1.11)		.59
Right ventriculotomy	1.98 (1.14-3.43)		.015	1.01 (0.47-2.18)		.99
PVS procedure	0.39 (0.26-0.59)		<.001	0.47 (0.23-0.94)		.033

*HR*, Hazard ratio; *CI*, confidence interval; *PVS*, pulmonary valve-sparing.

### Outcomes After Tetralogy of Fallot Repair by Pulmonary Valve Sparing

**Figure E2:**
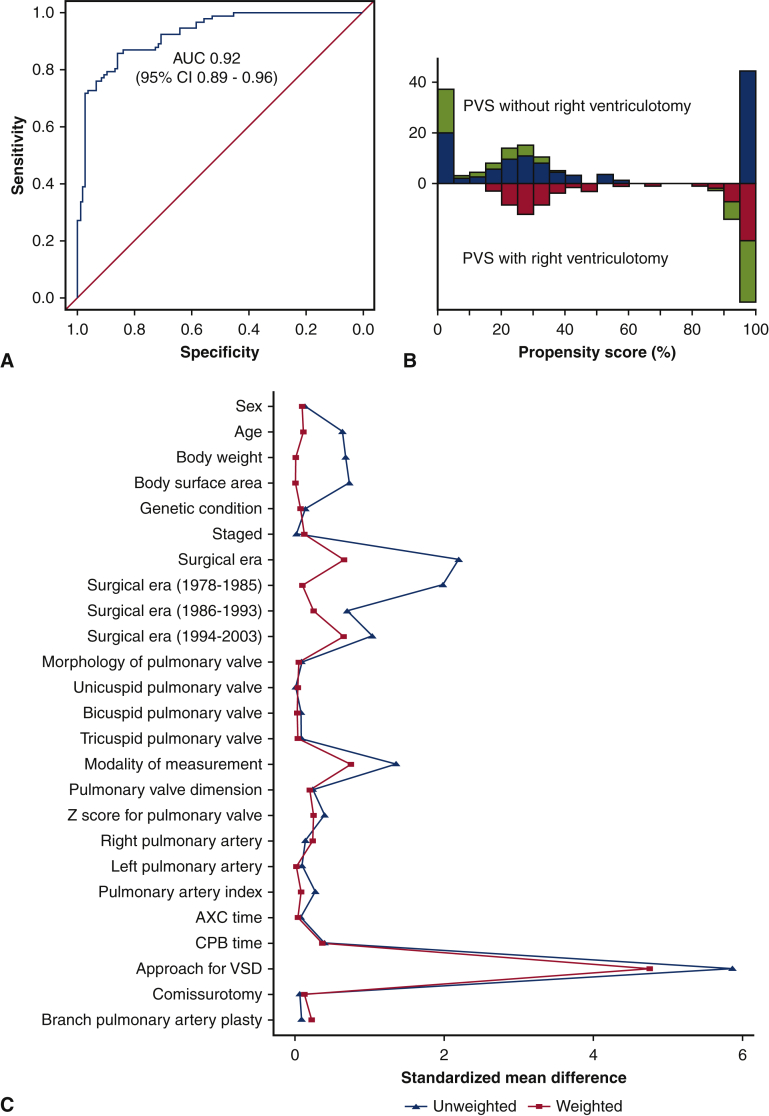
A, Receiver operating characteristic curve of the propensity score for the PVS group. B, Distribution of propensity score among the PVS patients in weighted (*colored*) and unweighted (*white*) cohorts. C, Covariable balance plot of PVS patients. *AUC*, Area under the curve; *CI*, confidence interval; *PVS*, pulmonary valve-sparing; *AXC*, aortic crossclamping; *CPB*, cardiopulmonary bypass; *VSD*, ventricular septal defect.

The area under the curve for the propensity score to undergo right ventriculotomy was 0.92 (95% CI, 0.89-0.96) (Figure E2, *A*). The distribution of propensity score is shown in Figure E2, *B*. Table 3 presents the characteristics of the patients who underwent the PVS procedure.

**Table 3:** Patient characteristics of pulmonary valve-sparing procedures

Group	Unweighted cohort			Weighted pseudo-cohort		
	Ventriculotomy (-)	Ventriculotomy (+)	SMD	Ventricultomy (-)	Ventriculotomy (+)	SMD
N	106	92		113.5	72.1	
Female	45 (42.5)	33 (35.9)	0.135	43.1 (38.0)	24.0 (33.4)	0.096
Age at repair (y)	1.9 ± 1.4	2.9 ± 1.8	0.636	2.5 ± 1.8	2.6 ± 1.6	0.113
Body weight (kg)	9.8 ± 2.9	11.9 ± 3.3	0.682	11.5 ± 3.5	11.5 ± 2.8	0.010
Body surface area (m^2^)	0.45 ± 0.10	0.53 ± 0.11	0.730	0.51 ± 0.12	0.51 ± 0.10	0.004
Genetic condition	5 (4.7)	2 (2.2)	0.140	2.8 (2.5)	1.1 (1.5)	0.073
Staged repair	11 (10.4)	9 (9.8)	0.020	7.4 (6.5)	7.2 (10.0)	0.126
Surgical era			2.196			0.654
1978-1985	3 (2.8)	65 (70.7)	1.979	44.1 (38.8)	31.6 (43.9)	0.102
1986-1993	66 (62.3)	27 (29.3)	0.700	49.6 (43.7)	40.5 (56.1)	0.251
1994-2003	37 (34.9)	0 (0.0)	1.036	19.8 (17.5)	0.0 (0.0)	0.650
Morphology of pulmonary valve			0.088			0.050
Unicuspid	1 (0.9)	1 (1.1)	0.014	1.2 (1.0)	1.0 (1.4)	0.034
Bicuspid	72 (67.9)	66 (71.7)	0.083	77.3 (68.1)	50.2 (69.5)	0.030
Tricuspid	33 (31.1)	25 (27.2)	0.087	35.0 (30.9)	21.0 (29.1)	0.039
Pulmonary artery measurements						
Modality			1.355			0.746
Catheter angiography	75 (70.8)	14 (15.2)		65.7 (57.9)	17.0 (23.5)	
Intraoperative measurement	31 (29.2)	78 (84.8)		47.8 (42.1)	55.2 (76.5)	
Pulmonary valve (mm)	12.3 ± 1.8	12.8 ± 2.8	0.239	13.4 ± 3.4	12.8 ± 2.5	0.197
Z score for pulmonary valve	0.28 ± 0.88	-0.14 ± 1.24	0.397	0.26 ± 1.06	-0.01 ± 1.14	0.246
Right pulmonary artery (mm)	9.4 ± 1.7	9.6 ± 1.8	0.139	9.2 ± 1.5	9.5 ± 1.7	0.236
Left pulmonary artery (mm)	8.7 ± 2.2	8.9 ± 2.2	0.093	8.7 ± 1.9	8.7 ± 2.0	0.018
Pulmonary artery index (mm^2^/m^2^)	309 ± 100	284 ± 81	0.274	267 ± 98	277 ± 74	0.079
Perioperative characteristics						
AXC time (min)	86 ± 23	88 ± 19	0.076	86 ± 24	85 ± 18	0.040
CBP time (min)	152 ± 39	167 ± 37	0.395	154 ± 43	169 ± 39	0.364
Approach for VSD			5.865			4.754
Right ventricle	0 (0.0)	86 (94.5)		0 (0)	65.1 (91.9)	
Right atrium/pulmonary artery	105 (100)	5 (5.5)		113.0 (100)	5.8 (8.1)	
Comissurotomy	89 (84.8)	76 (82.6)	0.058	89.3 (78.6)	60.4 (83.7)	0.129
Branch pulmonary artery plasty	2 (1.9)	3 (3.3)	0.087	1.9 (1.7)	4.3 (5.9)	0.223

Data represented as number (%) or mean ± standard deviation. *SMD*, Standardized mean difference; *AXC*, aortic crossclamping; *CPB*, cardiopulmonary bypass; *VSD*, ventricular septal defect.

**Figure 3:**
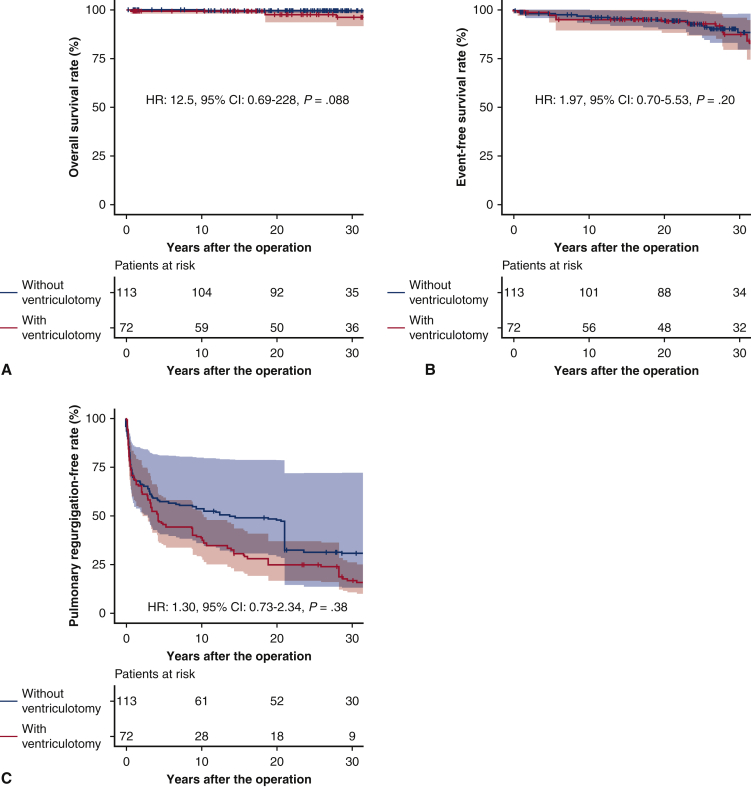
Overall survival (A), event-free survival (B), and moderate or greater pulmonary regurgitation-free rates (C) in the weighted cohort of the PVS group. *Bands above and below the fitted line* represented 95% CIs. *HR*, Hazard ratio; *CI*, confidence interval.

In the weighted cohort, the overall survivals at 10, 20, and 30 years were 100%, 99.5%, and 99.5% in the PVS without ventriculotomy group, and 99.3%, 97.4%, and 96.2% in the PVS with ventriculotomy group, respectively (HR, 12.5; 95% CI, 0.69-228; *P* = .088) (Figure 3, *A*). Event-free survivals at 10, 20, and 30 years were 96.6%, 94.4%, and 88.3% in the PVS without ventriculotomy group, and 94.8%, 93.8%, and 87.3% in the PVS with ventriculotomy group, respectively (HR, 1.97; 95% CI, 0.70-5.53; *P* = .20) (Figure 3, *B*). The moderate or greater pulmonary regurgitation-free survivals at 10, 20, and 30 years were 53.6%, 47.9%, and 30.8% in the PVS without ventriculotomy group, and 39.4%, 24.8%, and 16.8% in the PVS with ventriculotomy group, respectively (HR, 1.30; 95% CI, 0.73-2.34; *P* = .38) (Figure 3, *C*).

A recent follow-up of patients in the PVS with right ventriculotomy group showed that they have higher New York Heart Association grade (*P* = .007), larger cardiothoracic ratio on chest x-ray (*P* = .001), and higher rate of medication use (*P* = .019) (Table 4). A complete right bundle branch block occurred more frequently in the PVS without ventriculotomy group (*P* = .009). Additionally, the peak blood flow velocity across the right ventricular outflow tract was lower in the PVS with ventriculotomy group (*P* = .002).

**Table 4:** Comparison of clinical outcomes in pulmonary valve-sparing cases after inverse probability weighting

Group	Ventriculotomy (-)	Ventriculotomy (+)	*P*
No. in pseudo cohort	113.5	72.1	
From TOF repair (y)	26.2 ± 10.1	24.5 ± 12.7	.54
Mortality	0.5 (0.5)	2.4 (3.4)	.19
Atrial arrhythmia	15.6 (13.8)	7.8 (10.9)	.57
Ventricular arrhythmia	2.6 (2.3)	5.5 (7.7)	.097
Permanent pacemaker	1.3 (1.1)	2.0 (2.7)	.43
Pulmonary valve replacement	0.7 (0.6)	1.7 (2.3)	.36
Redo RVOT reconstruction	3.6 (3.1)	1.7 (2.3)	.74
New York Heart Association functional classification			.007
I	101.6 (89.3)	53.4 (73.6)	
II	11.9 (10.6)	18.8 (26.4)	
Cardiothoracic ratio (%)	47.4 ± 7.1	53.4 ± 7.9	.001
Brain natriuretic peptide (pg/mL)	37.5 ± 30.4	47.1 ± 46.6	.19
Medication			
Diuretics	3.0 (2.7)	5.9 (8.2)	.10
Beta-blocker	3.6 (3.2)	7.8 (10.8)	.047
ACEI/ARB	3.6 (3.1)	4.9 (6.8)	.26
Antiarrhythmic	0.5 (0.5)	0.5 (0.7)	.85
Medication free	107.4 (94.6)	60.4 (83.7)	.019
Recent electrocardiogram			
Duration of the QRS complex (msec)	131 ± 25	130 ± 30	.85
Complete right bundle branch block	65.5 (57.7)	27.3 (37.9)	.009
Results of recent transthoracic echocardiography			
Left ventricular ejection fraction (%)	69.8 ± 8.6	67.3 ± 12.0	.27
Right ventricular end-diastolic dimension (mm)	26.1 ± 6.7	28.0 ± 7.2	.26
Pulmonary regurgitation			
Trivial	19.5 (17.2)	7.3 (10.1)	.19
Mild	35.2 (31.1)	29.4 (40.8)	.18
Moderate	39.4 (34.7)	23.1 (32.1)	.71
Severe	19.3 (17.0)	12.2 (17.0)	1.00
Tricuspid regurgitation mild or more	78.9 (69.5)	42.5 (58.9)	.14
Peak velocity at pulmonary valve (m/s)	1.84 ± 0.53	2.00 ± 0.58	.14
Peak velocity at RVOT (m/s)	1.48 ± 0.51	1.10 ± 0.47	.002
Tricuspid annular plane systolic excursion (mm)	18.1 ± 2.8	18.3 ± 3.2	.80
End-diastolic forward flow	59.1 (52.0)	29.7 (41.1)	.15

Data represented as number (%) or mean ± standard deviation. *TOF*, Tetralogy of Fallot; *RVOT*, right ventricular outflow tract; *ACEI*, angiotensin-converting enzyme inhibitor; *ARB*, angiotensin II receptor blocker.

## Discussion

On propensity score matching, only a small percentage of matched patients could be included in the analysis, but inverse probability weighting theoretically allows the entire study cohort to be incorporated in the comparative study. Each patient is assigned a weight that is the inverse of the propensity score, that is, a patient with a propensity score of 0.8 is treated as 1.25 patients. The standardized mean difference of a few weighted preoperative characteristics, such as approach for VSD closure, surgical era, and cardiopulmonary bypass time, exceeded 0.25. However, the standardized mean difference in the remaining preoperative and perioperative variables, namely, body weight, probability of genetic disorder, frequency of palliative shunt, pulmonary valve morphology, frequency of concomitant branch pulmonary artery plasty, and size of pulmonary valve or branch pulmonary artery, was less than 0.25.^14^ Thus, we believe that most of the considerable preoperative and perioperative confounders that may affect long-term outcomes were statistically well balanced after weighting such that we can compare outcomes accurately.

The PVS group demonstrated significantly better overall survival, event-free survival, and moderate or greater pulmonary regurgitation-free rates compared with the TAP group. Multivariate analysis determined that the PVS procedure had an independent protective effect against late adverse events. More than half of patients in both the TAP and PVS groups developed moderate or greater pulmonary regurgitation within 10 years, but pulmonary regurgitation progressed more significantly and rapidly in the TAP group; therefore, deterioration of pulmonary valve function should be one of the reasons why the TAP group showed inferior long-term outcomes.^6^

Comparing the PVS with and without ventriculotomy groups, no significant difference was observed in overall outcomes such as survival, event-free survival, and moderate or greater pulmonary regurgitation-free rates. Anatomically, the right ventricular free wall does not play as an important a role as the sinus and infundibulum do.^18^ So a minor incision on the free wall may not cause global right ventricular dysfunction. Regarding a macro-reentrant circuit for ventricular tachycardia, patch augmentation of the adequately sized outflow tract incision does not create a so-called isthmus 2, muscle substrate between the nadir of anterior semilunar leaflet of pulmonary valve and the outflow patch, or a so-called isthmus 1, muscle substrate between the tricuspid valve annulus and the patch, if the patch is attached to the nadir of anterior semilunar leaflet of pulmonary valve and placed far from the tricuspid annulus.^19^ Of course, late ventricular tachycardia can also originate from a so-called isthmus 3, muscle substrate at the infundibulum between the nadir of the right semilunar leaflet of the pulmonary valve and VSD patch. However, these probabilities are not thought to be different between PVS with and without right ventriculotomy.

**Figure 4:**
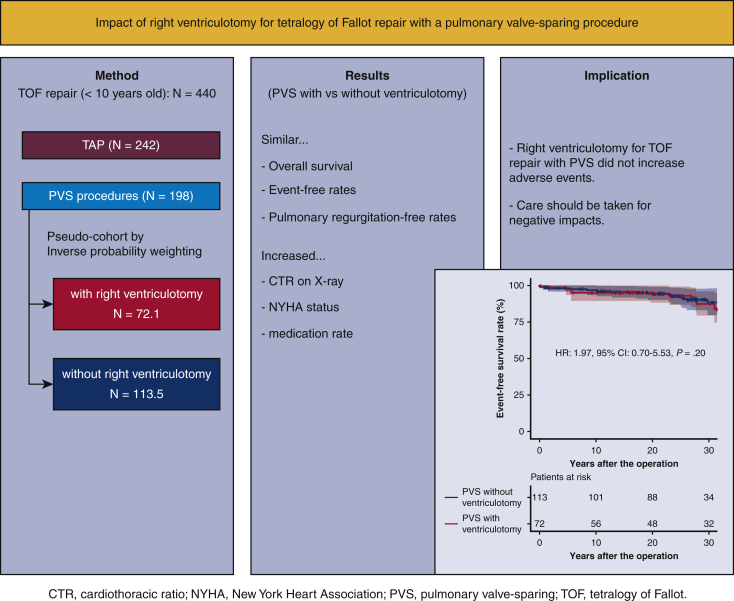
The comparison between TOF repair by PVS with and without right ventriculotomy showed no significant differences in overall survival, event-free survival, and pulmonary valve regurgitation-free rates, but the PVS without right ventriculotomy group had a smaller cardiothoracic ratio (*CTR*), superior New York Heart Association (*NYHA*) functional status, and less medication. *TOF*, Tetralogy of Fallot; *PVS*, pulmonary valve sparing; *TAP*, transannular patch.

However, excessive ventriculotomy causes later right ventricular dysfunction and ventricular arrhythmia.^4^ Scarring, aneurysmal dilatation, and a paradoxically moving or uncontractile outflow patch are known to be risk factors for right heart dilatation, low ejection fraction, and poor exercise tolerance.^10^, ^11^, ^12^ Indeed, a previous report has shown that those with TAP and PVS with right ventriculotomy similarly developed right ventricular dilatation and pulmonary valve insufficiency later,^20^ which indicated that avoiding right ventriculotomy was more advantageous than preserving the marginally small native pulmonary valve leaflets and annulus to provide better long-term arrhythmia-free survival. Although clinically insignificant thus far, the observed statistically significant difference in New York Heart Association functional status, medication-free rate, and cardiothoracic ratio might be derived from the unfavorable effects of a right ventriculotomy and an outflow patch (Figure 4). We emphasize again that right ventriculotomy should not be avoided if the pulmonary valve can be spared. This is because PVS was an independent predictor of the avoidance of adverse events. Moreover, further long-term scheduled follow-up is mandatory to identify whether the inferior clinical status of PVS in patients with right ventriculotomy, which is not significant to date, may be significant as more adverse events emerge later.

A previous study has shown that mildly persistent right ventricular outflow tract obstruction has a protective effect against the need for late PVR in the presence of significant pulmonary regurgitation.^21^ It is unclear, however, whether the statistically significant but clinically insignificant difference in pressure gradient across the right ventricular outflow tract (8.7 mm Hg in PVS without right ventriculotomy group vs 4.9 mm Hg in the PVS with right ventriculotomy group) is related to such differences in late clinical features.

The probability of a complete right bundle branch block was lower in the PVS with ventriculotomy group. Because the QRS duration did not differ in the 2 groups, its influence is thought to be clinically insignificant thus far. Moreover, a complete right bundle branch block after TOF repair is reported to impair ventricular function in itself,^22^^,^^23^ so the presence of statistically significant differences at long-term follow-up seems inconsistent. Complete right bundle branch block at TOF repair can occur through closure of the VSD, right ventriculotomy, or infundibulectomy; of these, an infundibulectomy via the transatrial approach is suspected to damage the right bundle branch specifically.^24^, ^25^, ^26^ In addition, the VSD was closed through a right ventriculotomy in 86 of 92 patients in the PVS with right ventriculotomy group (93.5%), which may have helped to reduce the occurrence of complete right bundle branch block.

### Study Limitations

The first limitation of this study was that the small number of events made the results of this comparative study difficult to interpret. Next, the Z score of pulmonary valve diameter of less than −2.0 was rare in patients undergoing PVS in this study cohort, which means that in the PVS procedure, the right ventricle may have been incised not to increase severe and tubular infundibular stenosis. The VSD was closed via a right ventriculotomy in the majority of patients in the PVS with right ventriculotomy group; thus, the right ventricle was not sacrificed to avoid TAP. Third, although the PVS procedure was identified as an independent predictor for the avoidance of late adverse events by multivariate Cox regression analysis, the sizes of PVD were less frequently recorded when PVDs were smaller. The 25th percentile of the Z value for PVD in the TAP group was −3.11, as shown in Table 1, although this is hard to believe because patients with more hypoplastic pulmonary valve annuli are expected to be more frequently included in the TAP group. Thus, the conducted risk analysis seems statistically correct, but results may be biased because of imperfect data collection. Fourth, the reason why the length of the right ventricular incision was unexpectedly longer than usual in the PVS with right ventriculotomy group (24 mm) was that the majority of patients in this group underwent VSD closure via right ventriculotomy. On the other hand, patient age at operation was older than the current standard (∼3 to 6 months); thus, the effects of a large right ventriculotomy may have been attenuated in our patient cohort. Fifth, although the number of pulmonary valve leaflets was noted in echocardiographic and operative reports, the tricuspid valve was excessively frequent in the PVS group, which might indicate that the PVS without right ventriculotomy group included some patients who should have been diagnosed with malalignment VSD with pulmonary stenosis, double-chambered right ventricle, or TOF-type double-outlet right ventricle. Unfortunately, echocardiography and cine angiogram were not available in most of our patients, many of whom underwent these operations more than 30 years ago. Finally, regional wall motion or fibrosis of the right ventricular outflow tract was not estimated by cardiac magnetic resonance imaging or histopathologic findings.

## Conclusions

PVS at TOF repair had a protective effect against occurrence of major adverse events. Right ventriculotomy in the TOF repair with PVS procedure did not increase major adverse events, but its negative impacts on recent clinical status cannot be ignored.

### Webcast

You can watch a Webcast of this AATS meeting presentation by going to: https://aats.blob.core.windows.net/media/21%20AM/AM21_C11/AM21_C11_01.mp4.

**Figure:**
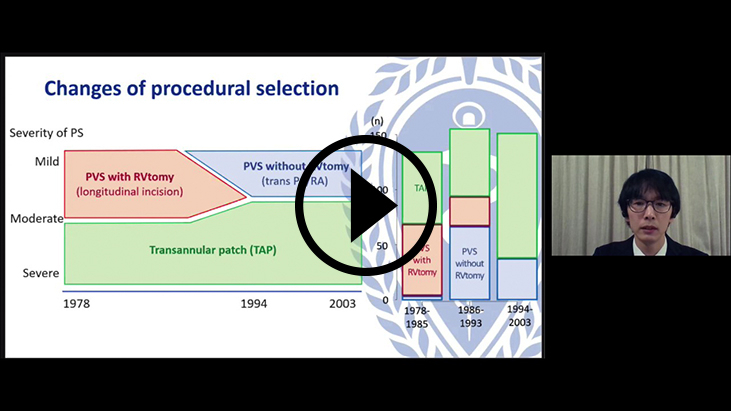

### Conflict of Interest Statement

The authors reported no conflicts of interest.

The *Journal* policy requires editors and reviewers to disclose conflicts of interest and to decline handling or reviewing manuscripts for which they may have a conflict of interest. The editors and reviewers of this article have no conflicts of interest.

## Supplementary Data

Video 1 Surgical procedures of PVS with and without right ventriculotomy. Video available at: https://www.jtcvs.org/article/S2666-2736(22)00005-5/fulltext.
