# Supplementary figures and images for: Impact of right ventriculotomy for tetralogy of Fallot repair with a pulmonary valve–sparing procedure
Source: JTCVS Open. 2022 Jan 22;9:191–205. doi: 10.1016/j.xjon.2021.10.061 (PMC9390402; doi:10.1016/j.xjon.2021.10.061)

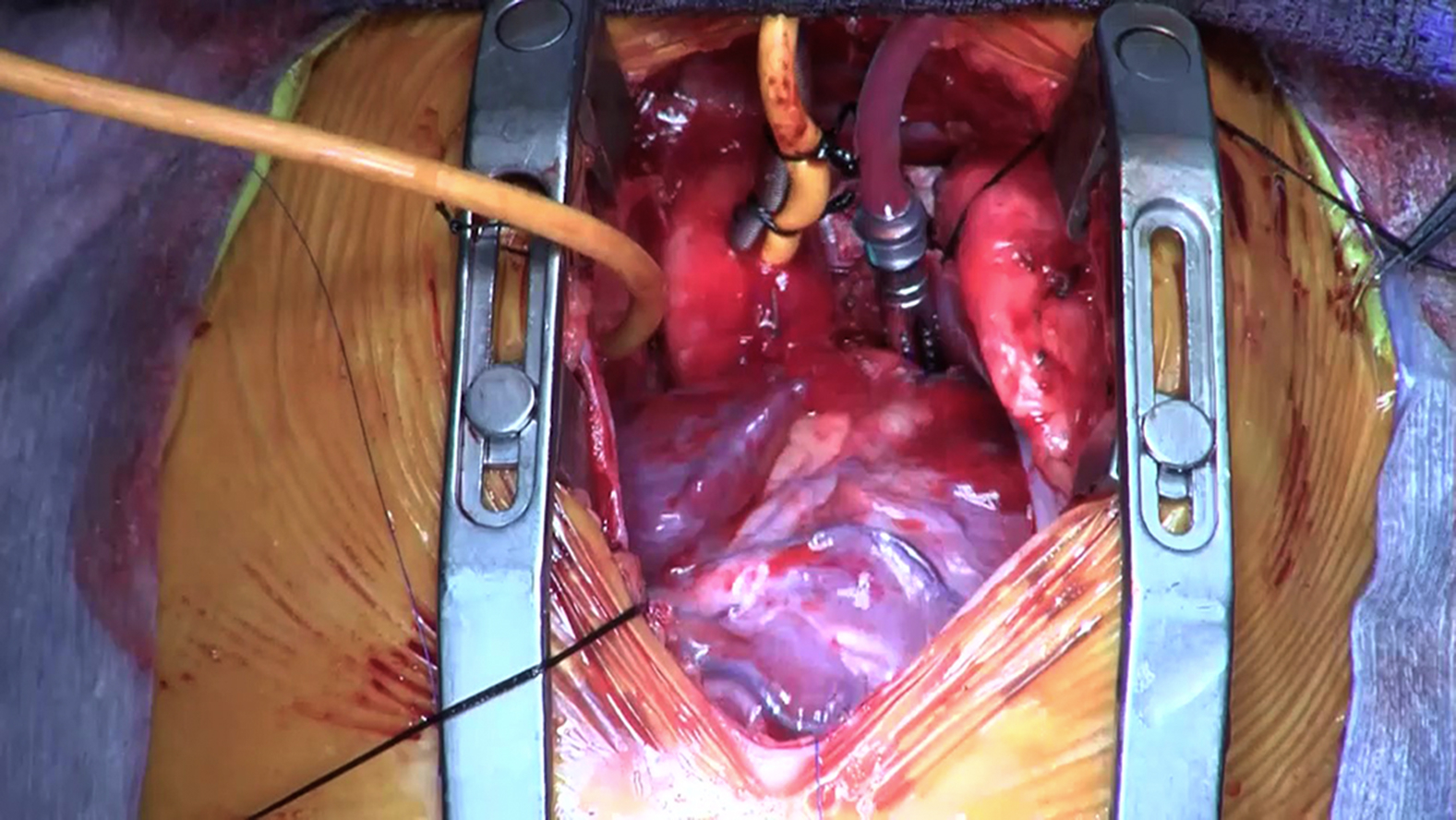

Supplement: Video 1 — Surgical procedures of PVS with and without right ventriculotomy. Video available at: https://www.jtcvs.org/article/S2666-2736(22)00005-5/fulltext. [file fx4.jpg]
